# Quality of Life in Cancer Patients Receiving Palliative Care

**DOI:** 10.4103/0973-1075.63133

**Published:** 2010

**Authors:** Divya Pal Singh

**Affiliations:** Home Care Service, Global Cancer Concern India, New Delhi, India

**Keywords:** Quality of Life, FACT-G^©^ questionnaire, Karnofsky performance status

## Abstract

**Background:**

The main focus of palliative care services is to improve the patient’s quality of life (QOL), which is defined as the subjective evaluation of life as a whole or the patient’s appraisal and satisfaction with their current level of functioning compared with what they perceive to be possible or ideal.

**Aims:**

In this prospective study we attempt to validate the Hindi version of a questionnaire designed by the functional assessment of chronic illness therapy (FACIT) measurement system; to measure the subjective QOL of cancer patients receiving home-based palliative care, determine ease of use of the questionnaire and correlate the QOL of these patients with the objective assessment of their Karnofsky’s performance status and their numerical pain score.

**Settings and Design:**

One hundred cancer patients receiving free home-based palliative care in New Delhi, India.

**Materials and Methods:**

A multidisciplinary palliative home care team using the Functional Assessment of Cancer Therapy-General (FACT-G^©^) questionnaire in Hindi.

**Statistical Analysis Used:**

Microsoft Excel Correlation.

**Results:**

The FACT-G^©^ questionnaire in Hindi is a useful tool in measuring QOL and can be used to monitor the patient’s progress and symptom control during the course of the disease. It is simple to use and does not take too much time to complete. The results are tabulated in English and can be used for comparison purposes globally; the scoring process is very simple.

**Conclusions:**

Increasing QOL and KPS showed a positive correlation whereas increasing pain and better QOL show negative correlation, as do better performance status and increasing pain score.

## INTRODUCTION

The main focus of palliative care services is to improve patient’s quality of life (QOL). The potential value of assessment of QOL in palliative care is being increasingly realized.[1] QOL is a subjective concept and its definitions and the sub-concepts involved have varied.

Safee *et al*, have defined QOL as the subjective evaluation of life as a whole or the patient’s appraisal and satisfaction with their current level of functioning compared with what they perceive to be possible or ideal.[2]

In a simple way, QOL is individual imagination or thought from a life style according to his/her objectives, expectations, standards and preferences. QOL is a multidimensional construct encompassing perceptions of both positive and negative aspects of dimensions such as physical, emotional, social and cognitive functions, as well as the negative aspects of somatic discomfort and other symptoms produced by a disease or its treatment.[3]

Slevin *et al*, maintain that to determine whether assessments of QOL by health professionals are meaningful and reliable, it is necessary to examine the correlation between the scores obtained by the health professionals and the final arbiters – the patients themselves.[4]

In an attempt to quantify QOL, many scales have been devised, revised and adapted over the years including (University of Wisconsin-Quality of Life) UW-QOL, European Organisation for Research and Treatment of Cancer- Quality of Life Quotient (EORTC QLQ-C30), and McGill QOL. The EORTC QLQ-C30 is available in Hindi, Bengali, Gujarati, Malayalam, Marathi, Punjabi, Tamil and Telugu; however, for this study, the FACIT system was preferred for its simple language and easy scoring system.

Questionnaires give a structured snapshot or insight into the patients’ point of view. They facilitate multi-disciplinary teams working with poor outcome groups, better information for the patients and their care givers, and the opportunity to identify problem areas and target intervention/support.[5]

Karnofsky Performance Status (KPS) is a standard way of measuring the ability of cancer patients to perform ordinary tasks. The scores range from 0 to 100. A higher score means the patient is better able to carry out daily activities. KPS may be used to determine a patient’s prognosis, to measure changes in a patient’s ability to function, or to decide if a patient could be included in a clinical trial. It is named after Dr. David A. Karnofsky, who described the scale with Dr. Joseph H. Burchenal in 1949.[6]

## MATERIALS AND METHODS

Our organization, Global Cancer Concern India, has a multidisciplinary team of a nurse, counselor and doctor offering free home-based palliative care to cancer patients. These patients are either referred by oncologists or they approach us directly. The patients selected for our prospective study were Hindi speaking (although their mother tongue may be different e.g., Punjabi, Rajasthani or Brajbhasha) and a mixture of adult males and females from a wide age range and socio-economic background. Many of them had advanced malignant disease, while some had recently been diagnosed. Many of the patients had received cytotoxic chemotherapy or radiotherapy at some point in their treatment.

Exclusion criteria: Those excluded from the study were pediatric patients, cognitively impaired, clinically depressed or withdrawn, with a Karnofsky’s Performance Status of 40 or less, or terminally ill (Palliative Prognostic Score of Risk group C, i.e., <30% chances of 30 day survival).

According to Tang physical or cognitive deterioration may hamper the ability or willingness of cancer patients to participate and remain in QOL research at the end of life. Use of family care-givers as proxy informants to report patients’ QOL has been suggested as a way of resolving the problem of non-response bias and non-random missing data,[7] but the nature of some questions, like those about sexuality, precluded this modality for patients who were very ill to answer on their own.

Before the interview, patients were explained the purpose of these questions and their participation was requested and a written or verbal consent obtained. Confidentiality was assured. None of the patients selected refused to participate in the study. The patients either answered the questions themselves or the counselor assisted them (especially the illiterate or the physically impaired); the nurse established the pain score using the Numerical Rating Scale (NRS), as the Brief Pain Inventory was considered too detailed and time consuming to be completed and scored. The doctor determined the performance status using the Karnofsky’s Performance Scale; as mentioned earlier, this scale measures the extent to which a patient’s symptoms restrict their activity and necessitate medical care.

**Figure 1:**
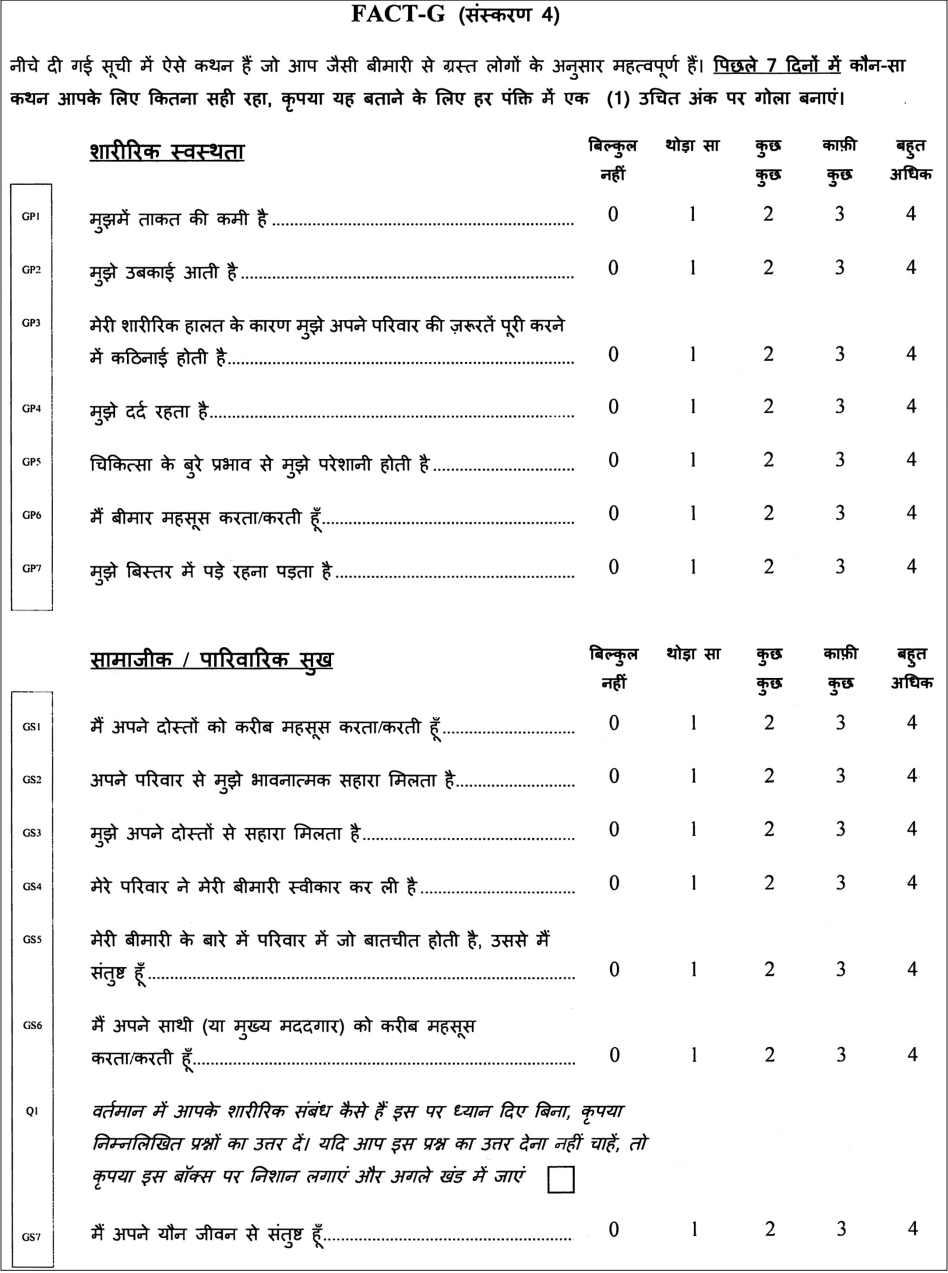
FACT-G Hindi quality of life questionnaire (Page 1)

**Figure 2:**
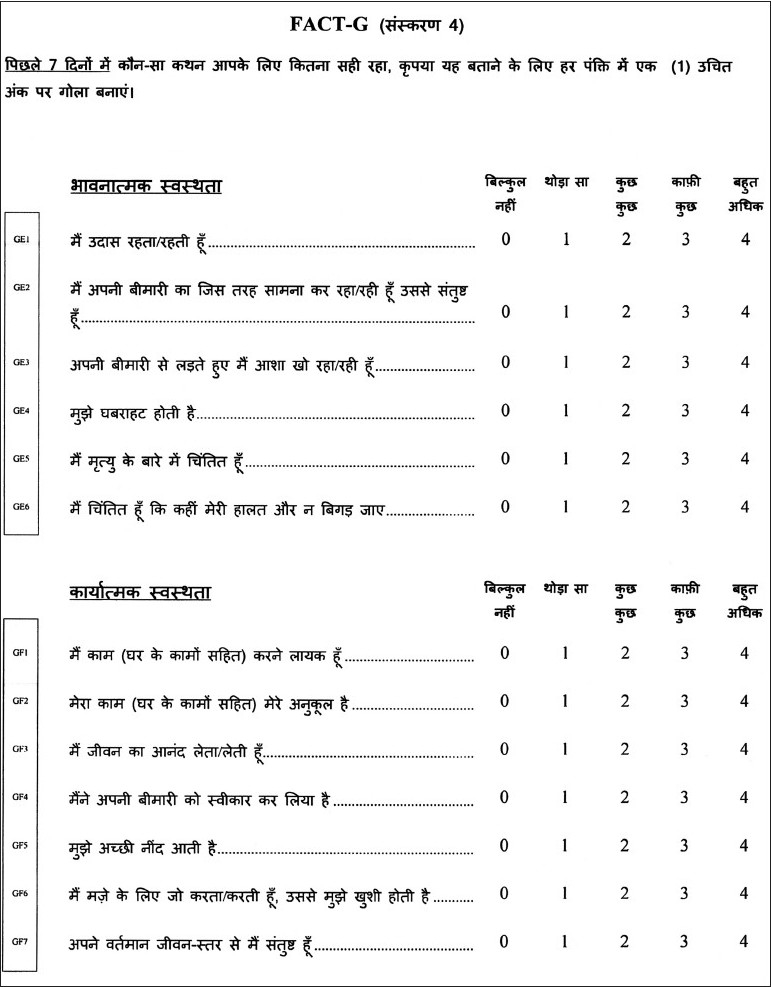
FACT-G Hindi quality of life questionnaire (Page 2)

**Figure 3:**
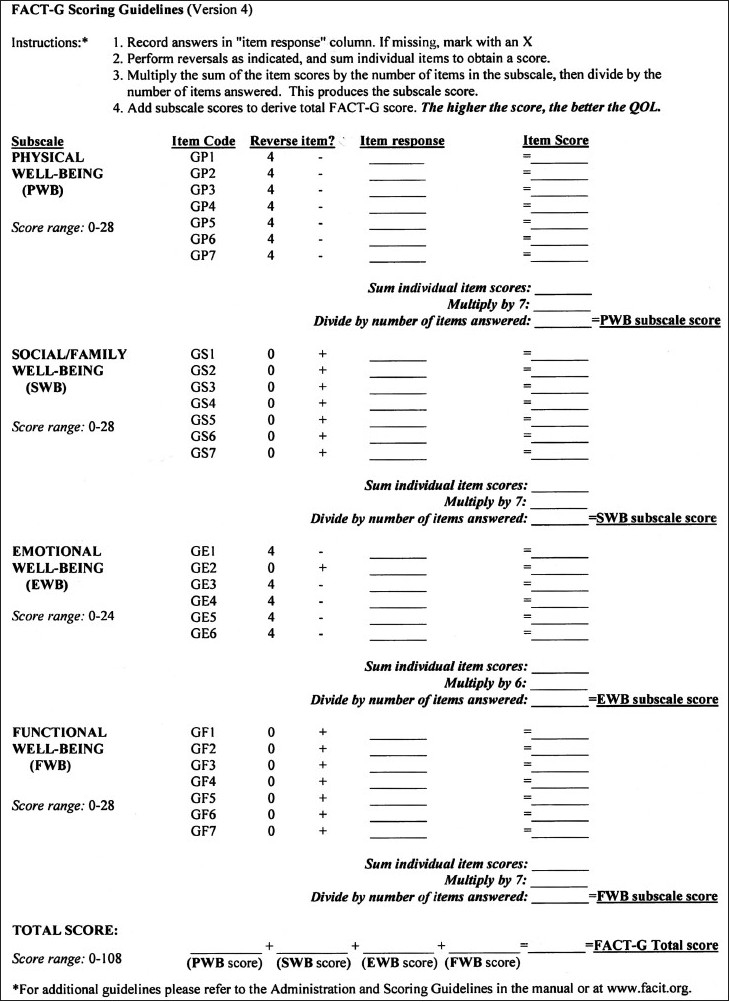
FACT-G Hindi quality of life questionnaire (Scoring Guidelines)

The functional assessment of chronic illness therapy system of QOL questionnaire (“FACIT^©^ System”) was designed by David Cella Ph.D. from FACIT.com, 381 S. Cottage Hill Avenue, Elmhurst, IL 60126, USA. The Hindi version of the FACT-G was licensed to me for use in this study [Figures 1 and 2]. The questionnaire has four sections: measuring Physical, Social/Family, Emotional and Functional well-being. The responses are then tabulated in a FACT-G Scoring Template [Figure 3] and the QOL obtained.

All surveys were done at the patients’ home. The study was conducted from Sep 2008 to May 2009. The total number of patients was 100. Average time taken to complete the questionnaire was six minutes; the average time taken to explain about the survey, getting consent and completing it was about 15 minutes. Sixty one per cent of the patients needed assistance in filling up the questionnaire.

The background of the patients may have varied, from the local Punjabis and Haryanvis, to migrants from Bengal, Bihar, UP, MP, Rajasthan, North Eastern and Southern states, but all had a basic understanding of Hindi and answered most of the questions fairly easily.

## RESULTS

Sex ratio: 63% were female and 37% male.

**Figure 4:**
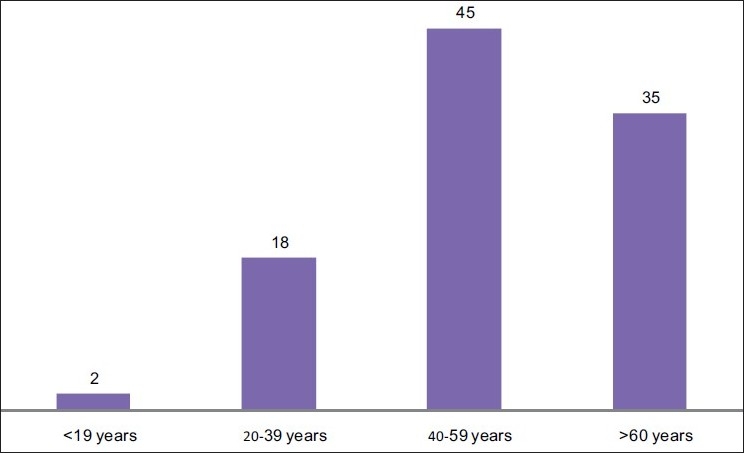
Age distribution

Age distribution: Most patients were middle-aged and elderly [Figure 4].

**Figure 5:**
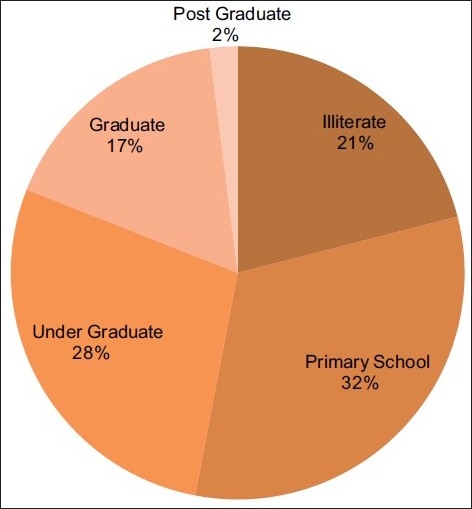
Literacy levels

Literacy levels: [Figure 5].

**Figure 6:**
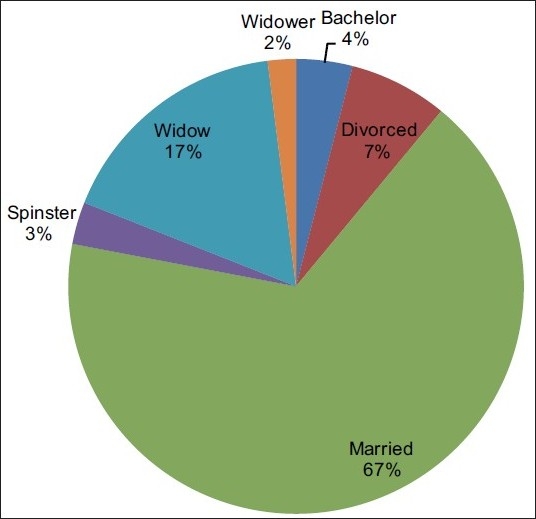
Marital status

Marital status: Most (67%) patients were married [Figure 6]. There was one couple where both partners had cancer – the husband had Ca Tongue whereas his wife had Ca Ovary.

**Figure 7:**
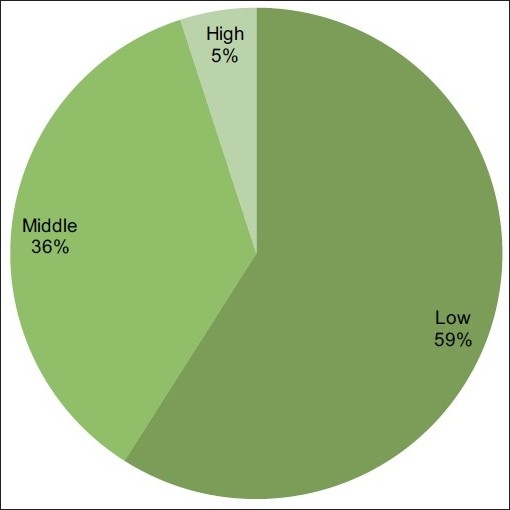
Socioeconomic status

Socio-economic status: According to the Modified Uday Pareek Scale, most of the patients (59%) were from a low socio-economic status [Figure 7].

**Figure 8:**
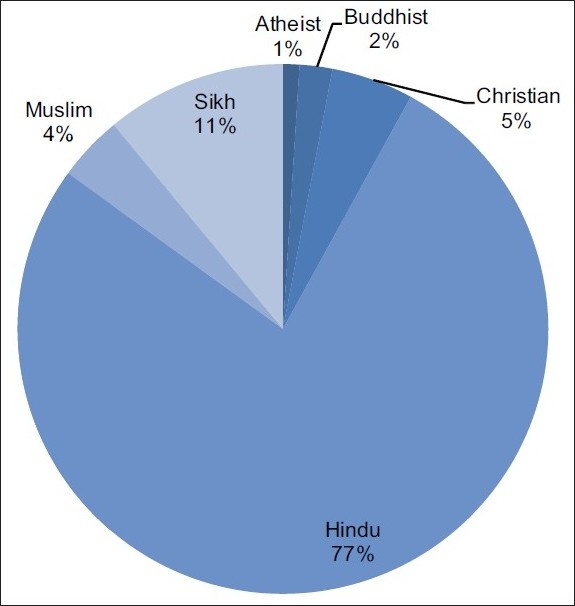
Religious background

Religion: Nearly 80% of the patients surveyed were Hindu; the other significant religion was Sikhism [Figure 8].

Concurrent diseases: 39% patients had concurrent diseases that affected the QOL [Table 1]. 3% had paraplegia as a consequence of cancer but only 2% had pressure sores and 9% had severe lymphoedema that adversely affected the patients’ performance status. 36% had various metastases and 5% had pathological fractures.

**Table 1:** Concurrent diseases

Hypertension	11	Chronic renal failure	2
Diabetes	5	Hyperthyroidism	2
Bronchial asthma	5	Blind	1
Anemia	4	Coronary artery disease	1
Osteoarthritis	3	Tuberculosis	1
Hypertension with diabetes	3	Epulis	1

Types of cancer: [Table 2].

**Table 2:** Types of cancers

Ca breast	20	Ca alveolus	2	Malignant melanoma	2
Ca cervix	12	Ca colon	2	Neurofibroma	2
Ca esophagus	7	Ca ovary	2	Non hodgkin’s lymphoma	2
Ca buccal mucosa	7	Ca prostate	2	Ca gall bladder	1
Ca lung	7	Ca thyroid	2	Ca para-nasal sinus	1
Ca larynx	5	Ca tonsil	2	Ca parotid gland	1
Ca tongue	4	Chronic myeloid leukemia	2	Ca stomach	1
Multiple myeloma	4	Ewing’s sarcoma	2	Hodgkin’s lymphoma	1
Metastasis of unknown origin	3	Glioblastoma multiforme	2	Malignant peripheral nerve sheath tumour	1
				Renal cell carcinoma	1

**Figure 9:**
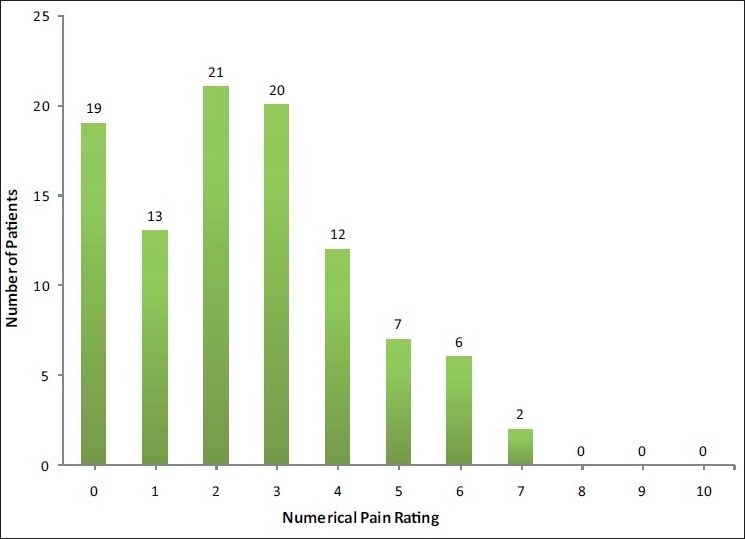
Numerical pain scores

Pain:19% patients had no pain. Most patients (54%) had mild pain. No patients had severe pain (Numerical Rating Scale of >8) [Figure 9].

**Figure 10:**
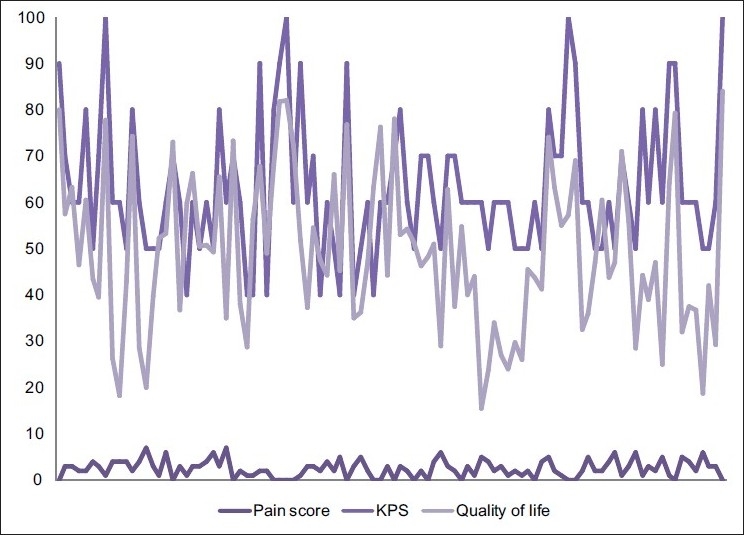
Raw data

When the raw data of the three parameters were represented graphically, they were random lines with no clear trend [Figure 10].

**Figure 11:**
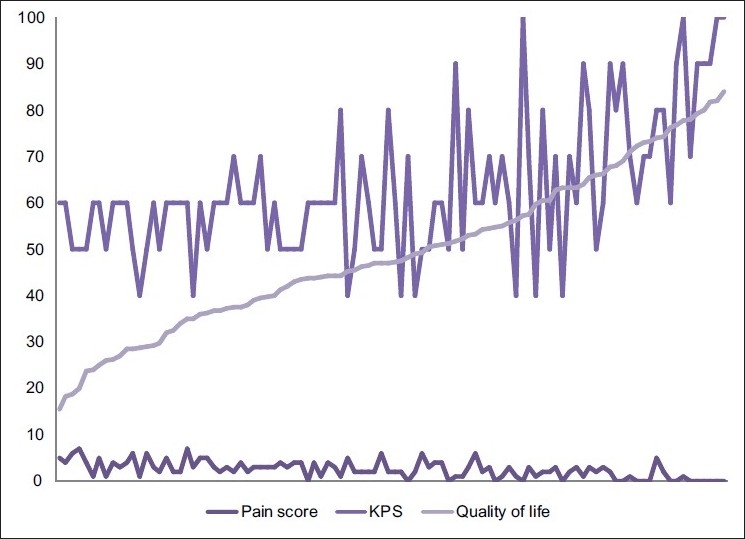
Graphical comparison of quality of life, Karnofsky performance status and numerical pain score

**Figure 12:**
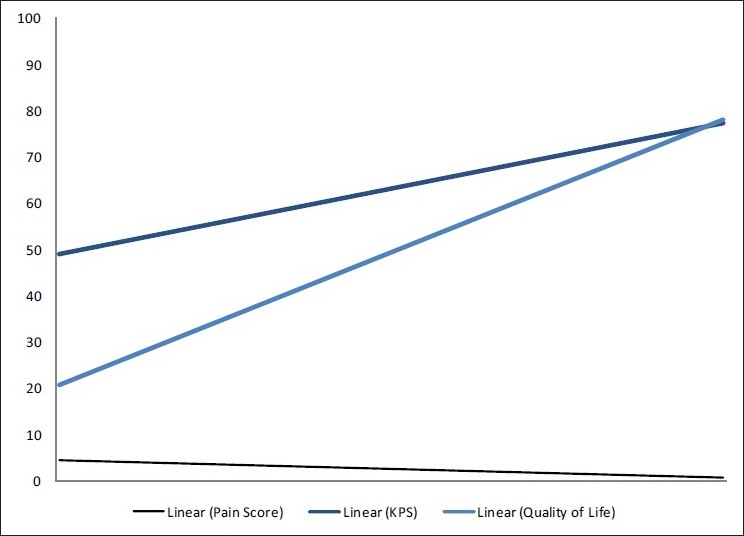
Trend lines of quality of life, Karnofsky performance status and numerical pain scores

However, when the QOL was put in ascending order, the corresponding KPS and Pain Score followed a similar trend [Figure 11]. The trend lines of KPS and the QOL both show an ascending trait [Figure 12] and the correlation is 0.545576. Increasing pain and better QOL show the obvious negative correlation of -0.58786, as do better Performance Status and increasing Pain Score (-0.4096).

## DISCUSSION

The FACT-G^©^ questionnaire in Hindi is a useful tool in measuring QOL and can be used to monitor the patient’s progress during the course of the disease. It is simple to use and does not take too much time to complete. The results are tabulated in English and can be used for comparison purposes globally; the scoring process is very simple.

1. Question number 2 *“My work (include work at home) is fulfilling”* in Functional Well Being section needs a better translation in Hindi.
2. Sexuality is a delicate subject in the Indian milieu and responses are very poor about it. Despite the importance of satisfactory sexual relationships in the QOL of the general population, this aspect is rarely addressed properly even in a ‘normal’ medical setting e.g., the loss of libido associated with the use of anti-hypertensive in men or post-menopausal loss of sexual interest in women is rarely clarified by patients and doctors. Thus, in a palliative care setting, the issue of sexuality is even more difficult to address – since the medical professionals and care givers are ill-equipped to resolve such dilemmas in a satisfactory manner.

According to Kaasa and Loge, during end-of-life care, spirituality and existential issues become more prominent, as well as family members’ perception of quality of care. Outcome measures in palliative care require constructs that reflect the specific goals of palliative care, such as improving QOL before death, symptom control, family support and satisfaction, as well as patients’ perceptions of ‘purpose’ and ‘meaning of life’. It is generally recommended that internationally developed and validated patient-rated multi-dimensional questionnaires should be used when assessing Health Related Quality of Life in research.[8]

The FACIT^©^ system has questionnaires that measure spiritual well being in patients with chronic illness. These are not available in Hindi at present and the translation process is long. Either we have to develop such tools in Indian languages or depend on translations from foreign languages that may not strictly follow the simple and spoken regional language for patients to comprehend easily and may need cultural validation.
